# HER2-Low Breast Cancer: Molecular Characteristics and Prognosis

**DOI:** 10.3390/cancers13112824

**Published:** 2021-06-05

**Authors:** Elisa Agostinetto, Mattia Rediti, Danai Fimereli, Véronique Debien, Martine Piccart, Philippe Aftimos, Christos Sotiriou, Evandro de Azambuja

**Affiliations:** 1 Academic Trials Promoting Team, Institut Jules Bordet, l’Université Libre de Bruxelles (U.L.B), 1000 Brussels, Belgium; veronique.debien@bordet.be (V.D.); evandro.azambuja@bordet.be (E.d.A.); 2 Humanitas Clinical and Research Center—IRCCS, Humanitas Cancer Center, Via Manzoni 56, 20089 Milan, Italy; 3 Department of Biomedical Sciences, Humanitas University, Via Rita Levi Montalcini 4, 20090 Milan, Italy; 4 Breast Cancer Translational Research Laboratory J.-C. Heuson, Institut Jules Bordet, Université Libre de Bruxelles, 1000 Brussels, Belgium; mattia.rediti@bordet.be (M.R.); danai.fimereli@bordet.be (D.F.); christos.sotiriou@bordet.be (C.S.); 5 Medical Oncology Department, Institut Jules Bordet, 1000 Brussels, Belgium; martine.piccart@bordet.be; 6 Clinical Trials Conduct Unit, Institut Jules Bordet—Université Libre de Bruxelles, 1000 Brussels, Belgium; philippe.aftimos@bordet.be

**Keywords:** HER2-low, breast cancer, PAM50, TCGA, prognosis

## Abstract

**Simple Summary:**

The dichotomous classification of HER2 status is experiencing a paradigm change, leading to the identification of the so-called “HER2-low” category. The aim of our retrospective, observational study was to characterize intrinsic PAM50 subtypes within HER2-low primary breast tumors extracted from The Cancer Genome Atlas (TCGA) dataset and to describe the prognostic impact of HER2-low on survival outcomes. We evaluated 804 breast cancers and we identified 410 HER2-low tumors (336 with positive hormonal receptor status (HR+) and 74 with negative HR status (HR−)). HER2-enriched tumors were more frequent in HER2-low/HR− and HER2-low/HR+ subtypes, compared to HER2-negative/HR− and HER2-negative/HR+ subtypes, respectively (13.7% versus 1.6% and 1.2% versus 0.5%, respectively). We observed no significant differences in prognosis between HER2-low subtypes and each non-HER2-low subtype when paired by HR status. Our characterization of PAM50 intrinsic subtypes within HER2-low breast cancer ultimately supports further investigation of new treatment strategies in the HER2-low category, with new promising drugs being tested in the context.

**Abstract:**

Background: We aimed to determine the distribution of intrinsic subtypes within HER2-low breast cancer (BC), and to describe the prognostic impact of HER2-low status on survival outcomes. Methods: This is a retrospective, observational study of primary BC extracted from The Cancer Genome Atlas dataset. We described the distribution of PAM50 intrinsic subtypes within HER2-low BC subtype according to hormonal receptor status (positive (HR+) and negative (HR−)). Secondly, we assessed the impact of HER2-low on survival outcomes (progression-free interval (PFI), disease-free interval (DFI), and overall survival (OS)). Results: We analyzed 804 primary BCs, including 410 (51%) HER2-low BCs (336 HR+ and 74 HR−). The proportion of HER2-enriched tumors was higher in the HER2-low/HR− group compared to HER2-low/HR+ (13.7% versus 1.2%, respectively). HER2-enriched tumors were more frequent in HER2-low/HR− and HER2-low/HR+ subtypes, compared to HER2-negative/HR− and HER2-negative/HR+ subtypes, respectively (13.7% versus 1.6% and 1.2% versus 0.5%, respectively). We observed no significant differences in PFI, DFI, and OS between HER2-low subtypes and each non-HER2-low subtype paired by HR status. Conclusions: Our characterization of PAM50 intrinsic subtypes within HER2-low breast cancer may explain the different clinical behaviors and responses to treatment, and ultimately support further investigation of new treatment strategies in the HER2-low category. Moreover, it highlights the importance of considering HR status in the HER2-low category.

## 1. Introduction

The characterization of the Human Epidermal Growth Factor Receptor-2 (HER2) in breast cancer (BC) has dramatically evolved over the last three decades, from a biomarker of poor prognosis to one of clinical benefit for trastuzumab and other anti-HER2 agents, when overexpressed/amplified in tumor cells [1,2,3,4,5]. In clinical practice, international guidelines recommend a binary distinction between HER2-positive (HER2+) and HER2-negative (HER2−) BCs, to guide physicians’ treatment decisions [6]. However, a relevant proportion (≈45–55%) of tumors that are classified as HER2− are in fact what is now called HER2-low [7,8,9]. The HER2-low BC subtype represents a new recently proposed nomenclature for those tumors with an immunohistochemistry (IHC) assay score of 1+ or 2+ but with negative in situ hybridization (ISH) assay [7,10]. In clinical practice, these tumors are classified as triple-negative BC (TNBC) or, if hormone receptors (HRs) are expressed, as luminal BC, thus being considered to not benefit from anti-HER2 treatments. HER2-low BCs comprise a majority of HR-positive (HR+) tumors (≈83%) [10,11]; however, little is known about their molecular characterization. Recently, Schettini et al. have presented the distribution of PAM50 intrinsic subtypes of a large retrospective cohort (*n* = 1576) of HER2-negative BCs extracted from databases pertaining to different studies [12]. Although this was the first molecular description of HER2-low BC in a large BC cohort, the authors pointed out several limitations of their work, including the combination of data from several different datasets, as well as the heterogeneity of the study population, which included a large proportion of metastatic patients. Moreover, in almost 30% of cases, the analyzed biospecimen was not the primary tumor. In order to provide complementary information and to shed further light on the molecular characteristics of HER2-low BC, we performed a retrospective analysis of primary BCs extracted from The Cancer Genome Atlas (TCGA) dataset. Moreover, we aimed to provide a comprehensive evaluation of HER2-low BCs’ prognosis by assessing survival outcomes of the above-mentioned cohort.

Indeed, despite the paucity of data available in HER2-low breast disease, being a rather new and quite unexplored field, there is a strong rationale that, also in HER2-low tumors, different intrinsic subtypes may explain different clinical behavior and response to treatments, as this has been already observed for other breast cancer subtypes (i.e., HER2-positive, HR-positive, HER2-negative and triple-negative breast cancers) [13,14,15].

## 2. Methods

### 2.1. Study Population and Data Extraction

We analyzed a retrospective cohort of primary BCs extracted from the publicly available breast cancer dataset generated by the TCGA Research Network (http://cancergenome.nih.gov/ (accessed on 15 October 2020)). Gene expression data (FPKM-UQ), PAM50 subtypes of primary tumor samples, and clinicopathological data were downloaded from the GDC Data Portal using TCGAbiolinks [16].

Only tumors with available HR and HER2 status by IHC were included in the present study.

Breast cancer subtypes were defined as follows: (i) HER2-low/HR+ (namely, HER2-low status and positive HR status), (ii) HER2-low/HR− (namely, HER2-low status and negative HR status), (iii) HER2+/HR+ (HER2-positive status and positive HR status), (iv) HER2+/HR− (HER2-positive status and negative HR status), (v) HER2−/HR+ (HER2-negative status and positive HR status), (vi) HER2−/HR− (HER2-negative status and HR negative status).

HER2 status was derived by IHC score/status and FISH status available in TCGA. In particular, information on HER2 status in TCGA dataset was evaluated according to three different forms provided: “HER2 IHC score”, “HER2 IHC status”, and “HER2 FISH status”. Tumors with a HER2 IHC assay score of 2+ were included only provided that HER2 status by FISH was available and not defined as equivocal. Tumors with an IHC assay score of 0, 1+ or 3+ could be included in the analysis even in absence of HER2 status by FISH. Tumors with HER2 IHC score “not available” could be included in the analysis, provided that HER2 IHC status was available. HER2-low status was defined as an IHC assay score of 1+ or 2+ without amplification at FISH assay, as defined in the clinical TCGA database.

Estrogen receptor (ER) and progesterone receptor (PgR) status were characterized according to the IHC data available. HR status was considered positive (HR+) when ER and/or PgR status was positive.

We included in the HER2−/HR− and HER2−/HR+ groups all tumors with HER2 IHC score of 0 as well as tumors with not available IHC score but defined as “negative” by IHC status and with FISH negative/not evaluated.

The “non-HER2-low” group was constituted by all breast tumors not matching the “HER2-low” definition, namely HER2-positive tumors as per ASCO-CAP definition (i.e., HER2 score 3+ at IHC and/or score 2+ with FISH amplification), as well as HER2-negative tumors, as defined above [17].

Intrinsic subtypes were assessed according to the PAM50 Breast Cancer Gene Signature Assay, which classifies BC subtypes in Luminal A, Luminal B, HER2-enriched, Basal-like, and Normal-like [18].

Curated survival data including overall survival (OS), progression-free interval (PFI), and disease-free interval (DFI) were obtained from the TCGA Pan-Cancer Clinical Data Resource (TCGA-CDR), developed by Liu et al. [19].

### 2.2. Study Objectives

Our primary objective was to characterize the molecular profile of HER2-low tumors included in our cohort of primary BCs extracted from TCGA dataset. Therefore, we characterized the distribution of PAM50 intrinsic subtypes within the HER2-low BC cohort, and we assessed the level of mRNA expression of *ERBB2* and *ESR1* genes in the different BC subtypes, including HER2-low tumors.

Our secondary objective was to describe the prognostic impact of HER2-low on patients’ outcomes. Therefore, we evaluated OS, PFI and DFI of HER2-low tumors compared to non-HER2-low tumors, and of HER2-low/HR+ tumors compared to HER2-low/HR− ones. OS was defined as the time from diagnosis until the date of death from any cause. PFI was defined as the time from diagnosis until the date of first occurrence of a new tumor event, which included disease progression, locoregional recurrence, distant metastasis, new primary tumor, or death with tumor. DFI was defined as the time from diagnosis until the date of the first new tumor event subsequent to the determination of a patient’s disease-free status after their initial diagnosis and treatment. Such event could be either a locoregional recurrence or a distant metastasis, the development of a new primary tumor in the same organ, or death from advancing of the same tumor. For PFI, unlike DFI, it was not necessary to first know whether a patient ever achieved disease-free status following their initial diagnosis and treatment.

### 2.3. Statistical Analysis

Statistical hypothesis tests involving the comparison of two or more groups were carried out using the Mann–Whitney U-test. False discovery rates (FDRs) were obtained using the Benjamini and Hochberg method. Survival data are shown using the Kaplan–Meier estimator, while the log-rank test was used to calculate the associated *p*-value. Fisher’s exact tests were used to evaluate associations between categorical data. Spearman correlation was used to determine the correlation between *ESR1* and *ERBB2* gene expression levels. All analyses were performed in R (version 4.0.2).

## 3. Results

### 3.1. Characteristics of Samples

Overall, we evaluated 1097 tumors from TCGA. Based on clinical and pathological criteria and available data, 804 tumors were selected for subsequent analyses (Figure S1 and Table S1). Pathological characteristics of samples included in the present analysis are reported in Table 1. We identified 410 (51%) HER2-low BC, of which 336 HR+ (41.8%) and 74 HR− (9.2%). A total of 133 tumors were defined as HER2+ BCs (16.5%), of which 101 HR+ (12.5%) and 32 HR− (4%), whereas 197 (24.5%) and 64 (8%) were classified as HER2−/HR+ and HER2−/HR−, respectively. PAM50 data were available for 789/804 tumors (98.1%), RNA-sequencing data for 799/804 (99.4%), and survival data for all 804 (100%) patients.

In order to ensure reliability of extracted data, we compared *ERBB2* gene expression levels according to HER2 IHC score (IHC score not available [NA] *versus* 0) in HER2−/HR− and in HER2−/HR+ tumors (Figure S2). No significant differences were noted for IHC score NA versus 0 in the HER2−/HR+ and HER2−/HR− groups (FDR = 0.769 and FDR = 0.239, respectively, *p*-values adjusted for the multiple testing within the HR+ or HR− group, separately, and FDR = 0.769 and FDR = 0.213, *p*-values adjusted merging HR+ and HR− groups). Moreover, we compared *ERBB2* gene expression levels according to HER2 IHC score in HER2-low/HR− versus HER2−/HR− tumors and in HER2-low/HR+ versus HER2−/HR+ tumors. Consistently, HER2−/HR+ and HER2−/HR− tumors with IHC score NA showed significantly lower *ERBB2* expression levels than those with IHC score of 1+ or 2+ in the HER2-low HR+ or HR− groups (FDR = 0.005 for IHC NA versus 1+, FDR < 0.001 for IHC NA versus 2+ in HR+ tumors; FDR = 0.003 for IHC NA versus 1+, FDR = 0.003 for IHC NA versus 2+ in HR− tumors; similar significant results were obtained when adjusting *p*-values in the HR+ and HR− groups together) (Figure S2).

### 3.2. Distribution of PAM50 Intrinsic Subtypes

**Figure 1:**
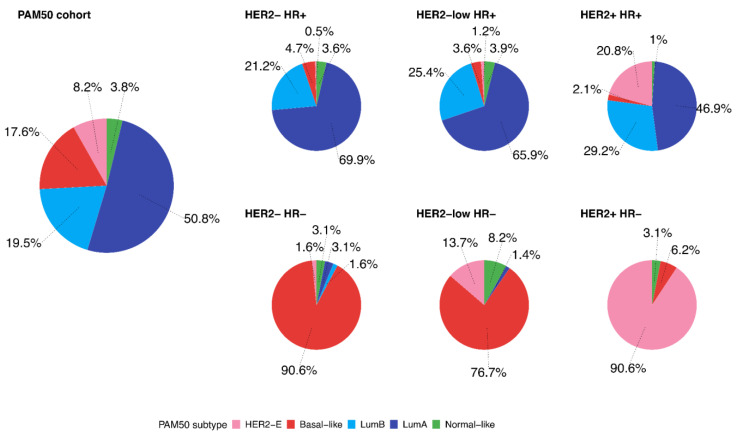
The distribution of PAM50 intrinsic subtypes in the entire cohort and among each breast cancer subtype (*n* = 789). Abbreviations: HR: hormone receptor, HER2-E: HER2-enriched, LumA: Luminal A, LumB: Luminal B.

Figure 1 shows the distribution of PAM50 intrinsic subtypes in the entire cohort (*n* = 789) and within each BC subtype. Overall, Luminal A was the most represented subtype (*n* = 401, 50.8%), followed by Luminal B (*n* = 154, 19.5%) and Basal-like tumors (*n* = 139, 17.6%). The distribution of the PAM50 subtypes significantly differed between the clinical groups (Table 2, *p* < 0.001, Fisher’s exact test with simulated *p*-value, based on 2000 replicates with Monte Carlo simulation). In particular, the distribution of PAM50 subtypes significantly differed between HER2−/HR− and HER2-low/HR− tumors (*p* = 0.015 at Fisher’s exact test), while the difference was not statistically significant when comparing HER2−/HR+ versus HER2-low/HR+ tumors (*p* = 0.738 for Fisher’s exact test).

HER2-low/HR− subtype was characterized by a higher proportion of HER2-enriched intrinsic subtypes compared to HER2−/HR− (13.7% versus 1.6%, respectively). Similarly, HER2-low/HR+ was characterized by a higher proportion of HER2-enriched intrinsic subtypes compared to HER2−/HR+ subtype (1.2% versus 0.5%), but the difference was less pronounced. As expected, both HER2-low/HR+ and HER2-low/HR− showed lower proportions of HER2-enriched intrinsic subtype compared to the HER2+/HR+ and HER2+/HR− subtypes (1.2%, 13.7%, 20.8%, and 90.6%, respectively).

**Figure 2:**
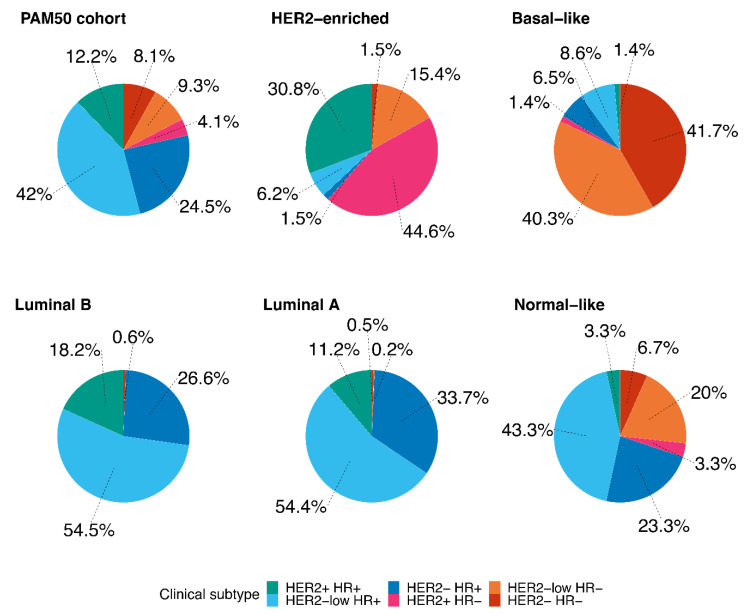
The distribution of breast cancer subtypes (HER2+/HR+, HER2-low/HR+, HER2−/HR+, HER2+/HR−, HER2-low/HR−, HER2−/HR−) according to PAM50 intrinsic subtypes (*n* = 789). Abbreviations: HR: hormone receptor.

Figure 2 shows the distribution of BC subtypes (HER2−/HR+, HER2+/HR+, HER2+/HR−, HER2-low/HR+, HER2-low/HR−, HER2−/HR−) according to the PAM50 intrinsic subtypes. In the HER2-enriched subgroup, HER2+/HR− and HER2+/HR+ subtypes were the most represented (44.6% and 30.8%, respectively); 6.2% and 15.4% of the HER2-enriched tumors were HER2-low/HR+ and HER2-low/HR−, respectively. Of note, HER2-low/HR− and HER2−/HR−, taken together, accounted for the great majority of Basal-like tumors (82%). Interestingly, the HER2-low/HR+ was the most represented subtype among Luminal A and Luminal B tumors (54.4% and 54.5%, respectively).

### 3.3. ERBB2 and ESR1 Gene Expression

**Figure 3:**
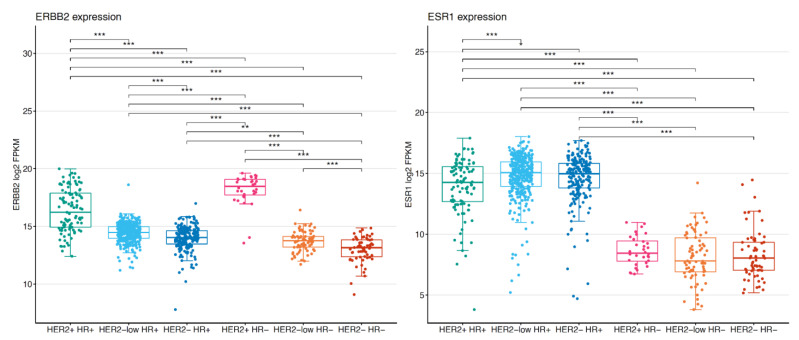
*ERBB2* and *ESR1* gene expression according to breast cancer subtypes (*n* = 799). In the boxplots, the boxes are defined by the 25th to 75th percentiles; the median is shown as a bold colored horizontal line. Statistical differences were assessed using a two-sided Mann–Whitney U-test followed by Benjamin and Hochberg correction for multiple testing. FDRs < 0.05 are shown. Abbreviations: * 0.05 > adjusted *p*-value ≥ 0.01, ** 0.01 > adjusted *p*-value ≥ 0.001, *** adjusted *p*-value < 0.001, HR: hormone receptor.

The level of mRNA expression of *ERBB2* and *ESR1* was evaluated in 799 tumor samples. *ERBB2* expression was higher in HER2+ subtypes, followed by HER2-low subtypes, which showed significantly higher levels compared to HER2-negative subtypes (Figure 3). HER2−/HR− tumors showed the lowest expression of *ERBB2*. Of interest, *ERBB2* expression was significantly higher in HER2-low/HR+ compared to HER2-low/HR− tumors. Regarding *ESR1*, its expression was higher in HR+ subtypes (namely, the HER2−/HR+, HER2+/HR+, and HER2-low/HR+) compared to HR− subtypes (HER2−/HR−, HER2+/HR−, and HER2-low/HR−), as expected. No significant differences were observed in *ESR1* expression between HER2-low/HR+ and HER2−/HR+ tumors, as well as between the HR− subtypes.

We further explored the correlation between *ERBB2* and *ESR1* gene expression levels according to HER2 status (i.e., negative, low, positive) (Figure S3). In an analysis including both HR+ and HR− tumors, a negative correlation was found in HER2+ tumors (Spearman = −0.46, *p* < 0.001), while both the HER2-low and HER2- groups presented a positive correlation between *ESR1* and *ERBB2* expression levels (Spearman 0.34, *p* < 0.001 and Spearman 0.38, *p* < 0.001 for HER2-low and HER2- tumors, respectively). The correlations were still significant although less pronounced when limiting the analysis to HR+ tumors (Spearman correlations of −0.29, 0.18, and 0.14 in HER2+, HER2-low and HER2- tumors, respectively), while no significant correlation (*p* > 0.05) was noted in HR− tumors.

### 3.4. Survival Analysis

Prognostic data of 804 patients were available and included in the present analysis.

**Figure 4:**
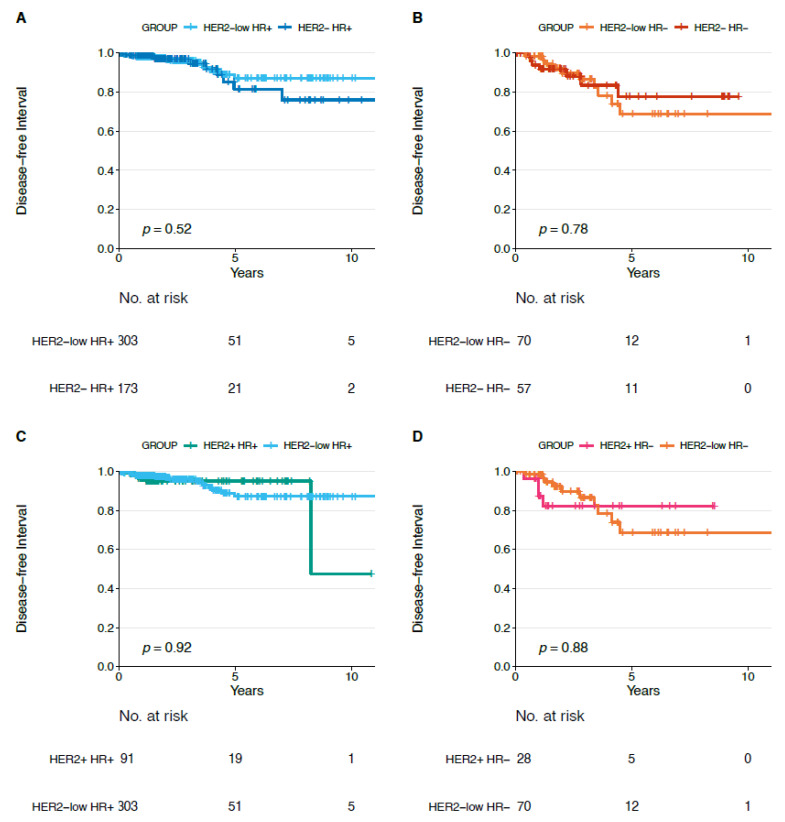
Disease-free interval in HER2-low/HR+ versus HER2−/HR+ (**A**), in HER2-low/HR− versus HER2−/HR− (**B**), in HER2-low/HR+ versus HER2+/HR+ (**C**) and in HER2-low/HR− versus HER2+/HR− (**D**) tumors. Abbreviations: HR: hormone receptor.

**Figure 5:**
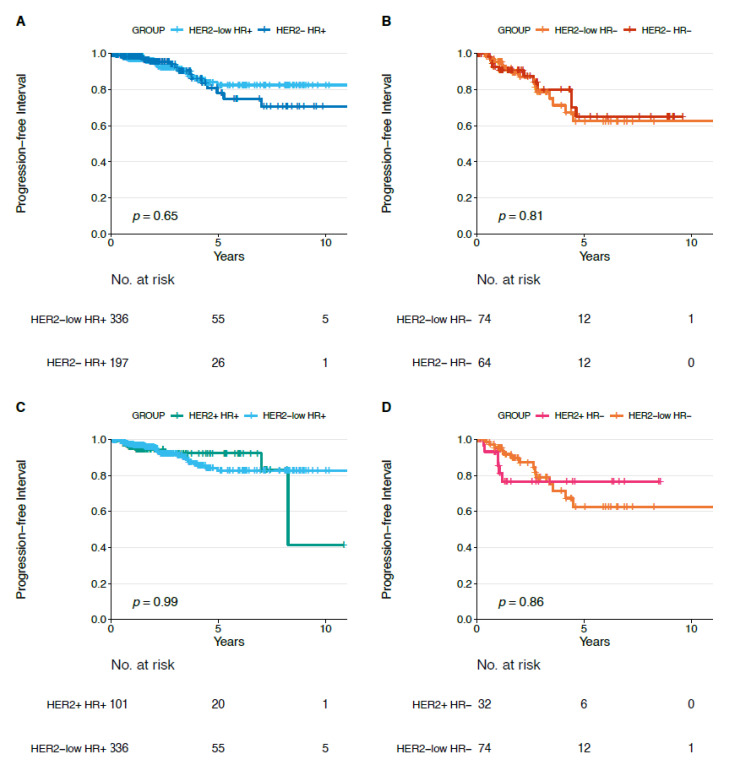
Progression-free interval in HER2-low/HR+ versus HER2−/HR+ (**A**), in HER2-low/HR− versus HER2−/HR− (**B**), in HER2-low/HR+ versus HER2+/HR+ (**C**) and in HER2-low/HR− versus HER2+/HR− (**D**) tumors. Abbreviations: HR: hormone receptor.

**Figure 6:**
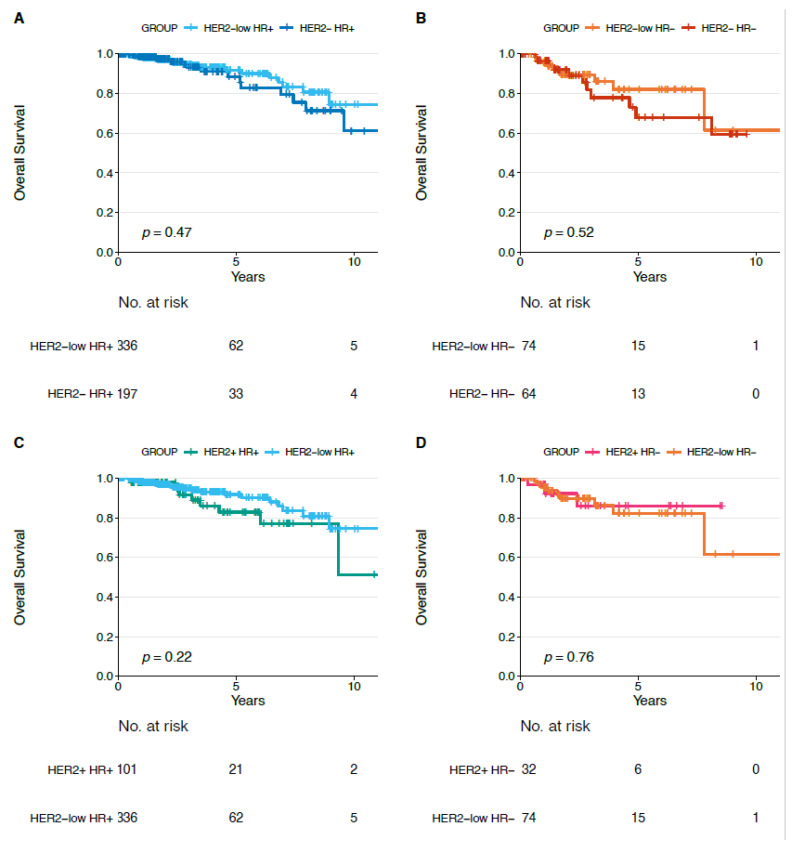
Overall survival in HER2-low/HR+ versus HER2−/HR+ (**A**), in HER2-low/HR− versus HER2−/HR− (**B**), in HER2-low/HR+ versus HER2+/HR+ (**C**) and in HER2-low/HR− versus HER2+/HR− (**D**) tumors. Abbreviations: HR: hormone receptor.

We did not observe statistically significant differences in terms of DFI, PFI, and OS for each HER2-low subtype (HR+ and HR−) compared to each non-HER2-low subtype (i.e., HER2−/HR−, HER2+/HR−, HER2−/HR+, HER2+/HR+) (Figure 4, Figure 5 and Figure 6).

**Figure 7:**
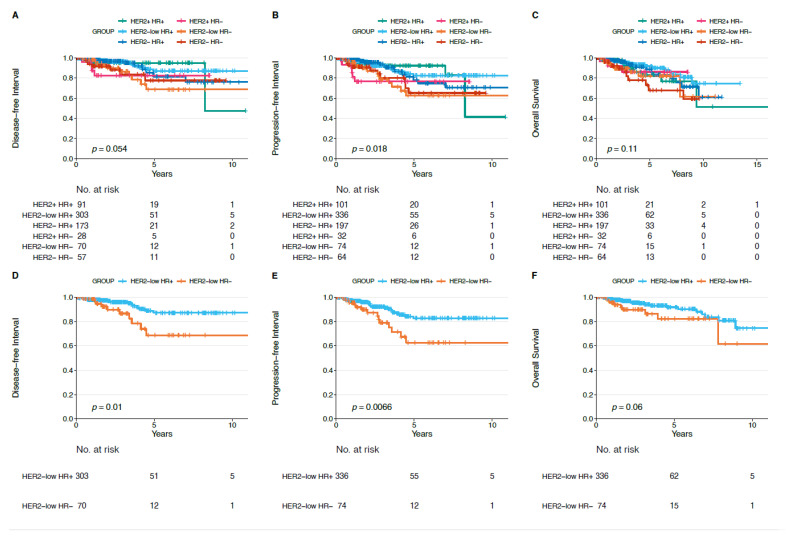
(**A**–**F**). Disease-free interval (DFI), progression-free interval (PFI), and overall survival (OS) according to breast cancer subtypes (**A**–**C**), and in HER2-low/HR+ versus HER2-low/HR− breast tumors (**D**–**F**). Abbreviations: HR: hormone receptor.

Figure 7 shows the Kaplan–Meier curves for DFI, PFI and OS for each BC subtype. Overall, there was a statistically significant difference (*p* = 0.018) (Figure 7B) in PFI among the different BC subtypes. Indeed, HER2−/HR− and HER2-low/HR− showed the worse outcome, with a 5-year PFI of approximately 60% (5-year PFI rate of 65% for HER2−/HR− and 63% for HER2-low/HR−). On the contrary, HER2−/HR+, HER2+/HR+ and HER2-low/HR+ were associated with better prognosis (5-year PFI rate of 92.3% in HER2+/HR+, 82.6% in HER2-low/HR+, and 78% in HER2−/HR+). Regarding DFI and OS, no significant differences (*p* = 0.054 and *p* = 0.11, respectively) were observed according to the different BC subtypes.

In addition, HER2-low/HR+ tumors showed better DFI and PFI when compared to HER2-low/HR− tumors (*p* = 0.01 and *p* = 0.0066, respectively) (Figure 7D,E).

Out of the 804 patients included in our analysis, 628 subjects had treatment information available in the TCGA dataset (279 HER2-low/HR+, 61 HER2-low/HR−, 23 HER2+/HR−, 75 HER2+/HR+, 144 HER2−/HR+, 46 HER2−/HR−) (Table S2). However, the treatment details were heterogeneous and incomplete, as previously reported [19]. Of note, according to the data available, the majority of patients with HER2+ tumors received an anti-HER2 treatment (56/98 HER2+ with treatment information, 40/75 HER2+/HR+ and 16/23 HER2+/HR−), while a small proportion of patients with HER2-low/HR+ (4/279), HER2-low/HR− (1/61) and HER2−/HR+ (2/144) tumors received HER2-targeting therapies as well. These data need to be interpreted with caution due to the lack of uniformity in the annotation of the treatments, and no conclusions regarding the impact of therapies on the patients’ prognoses in the different subtypes could be made.

## 4. Discussion

The dichotomous classification of HER2 status is experiencing a paradigm shift leading to the identification of the so-called “HER2-low” category, for which new treatment strategies are currently under evaluation [20,21,22,23,24,25]. The rising role of molecular profiling further helps in dissecting the heterogeneity within each BC subtype [13,26,27,28], including HER2-low BC [12]. In this work, we presented a retrospective analysis of molecular characteristics of primary HER2-low BC and a comprehensive analysis of its prognosis. In our cohort, HER2-low BC represented 51% of all tumors, with the majority (82%) being HR+; these findings are in line with previous reports [7,8,11]. When focusing on the distribution of PAM50 intrinsic subtypes in HER2-low tumors, we noticed that the distribution of intrinsic subtypes within HER2-low/HR+ BC was more similar to the one of HER2−/HR+ BC, rather than to HER2-low/HR− BC, and this is reassuring for clinical practice, since, thus far, HER2-low/HR+ BC are ascribed to HER2−/HR+ BC for the purpose of treatment. On the other hand, HER2-low/HR− BC showed a higher proportion of HER2-enriched intrinsic subtype, compared to HER2−/HR−. Hitherto, HER2-low/HR− BC is considered as HER2−/HR− in clinical practice, but this difference might reflect a dissimilar clinical behavior, and ultimately further supports the clinical investigation of new HER2-targeting agents in HER2-low BC. Antibody–drug conjugates (ADCs), such as trastuzumab deruxtecan and trastuzumab duocarmazine, have shown promising efficacy data in patients with metastatic HER2-low BC, attributed to the retention of all trastuzumab anti-tumor properties, associated with a bystander killing effect, which allows targeting and killing cancer cells even in tumors with lower degrees and heterogeneity of HER2 expression, a once limiting step for the clinical activity of anti-HER2 agents [20,21].

Furthermore, we presented the distribution of BC subtypes (HER2−/HR+, HER2+/HR+, HER2+/HR−, HER2-low/HR+, HER2-low/HR−, HER2−/HR−) according to PAM50 intrinsic subtypes. Interestingly, more than 50% of both Luminal A and B intrinsic subtypes were HER2-low/HR+ subtype, suggesting that in HER2-low/HR+ tumors, HR and “luminal” genes expression, and not HER2, might be the principal oncological driver. On the contrary, the HER2-low/HR− subtype was mainly represented in Basal-like intrinsic subtype (40.3%). The discrepancies existing between different classifications of breast tumors, according to pathological and molecular characteristics, may underline the need of a major integration of molecular information when selecting patients to maximize treatment benefit. Recent data on a biomarker analysis of intrinsic subtypes and efficacy across the MONALEESA trials showed that the HER2-enriched intrinsic subtype had the greatest reduction in the risk of progression and death after treatment with the CDK4/6 inhibitor ribociclib, compared to other intrinsic subtypes, while the Basal-like was the only subtype showing no benefit [15]. Taken together, these data support the role of molecular profiling as a possible strategy to dissect biological differences beyond the simple IHC classification of BC subtypes, and to better select patients who may benefit most from specific treatments.

We then evaluated the level of mRNA expression of *ERBB2* and *ESR1* according to BC subtypes. *ERBB2* expression was higher in HER2+ subtypes, followed by HER2-low subtypes, which showed significantly higher levels compared to HER2-negative subtypes. Moreover, *ERBB2* expression was higher in HER2-low/HR+ compared to HER2-low/HR− tumors. This latter observation is in apparent contrast with the fact that we observed no significant or pronounced enrichment for the HER2-enriched subtype in HER2-low/HR+ as compared to HER2-low/HR− by PAM50. However, a possible explanation resides in the fact that PAM50 subtyping is not only defined by *ERBB2* and *ESR1* expression levels. Our findings are consistent with those presented by Schettini et al. [12], with the authors suggesting that the lack of enrichment of the HER2-enriched subtype within the HER2-low disease may be explained based on previous evidence showing that the HER2-enriched phenotype is not defined by the sole expression of *ERBB2*. Indeed, it rather takes into account proliferation and *ESR1* expression levels as well [14]. Furthermore, despite the different expression profiling techniques used in the analysis of Schettini et al. and ours (Nanostring nCounter versus RNA-sequencing data, respectively), obtaining highly similar results further corroborates our findings. In addition, the two variables (i.e., HER2-enriched subtype and *ERBB2* levels) provide independent predictive and prognostic information [28,29].

In our analysis, *ESR1* expression was higher in HR+ subtypes (namely, HER2−/HR+, HER2+/HR+, and HER2-low/HR+), as expected, with no significant differences between HER2-low/HR+ and HER2−/HR+. The HR+ tumors were clearly dominated by a “luminal” phenotype, and the high expression of *ESR1* and other “luminal” genes may justify the lower proportion of HER2-enriched tumors observed in the HER2-low/HR+, despite the (marginally) higher *ERBB2* expression levels compared to the HER2-low/HR−.

Moreover, we observed an inverse correlation between *ERBB2* and *ESR1* gene expression levels in HER2-positive and -negative tumors (both when HR+ and HR− tumors were analyzed together and when limiting the analysis the the HR+ tumors), with results in line with previous reports [30]. In particular, HER2-low tumors behaved similarly to the HER2-negative ones, with a positive correlation between *ESR1* and *ERBB2* in the whole cohort including HR+ and HR− tumors, as well as in the HR+ subgroups, although less pronounced in the latter. Overall, these findings underline the need to separate the expression of single genes from the underlying tumor phenotype, and suggest that biological differences exist, beyond the simple distinction between HR+ and HR− tumors. Moreover, they can contribute to explain the different response rates observed for HER2-low/HR+ and HER2-low/HR− in clinical trials evaluating new ADCs in HER2-low metastatic BC [20,21]. For instance, in the phase I dose-escalation and dose-expansion study of trastuzumab duocarmazine, 9/32 (28%) patients with HER2-low/HR+ breast cancer and 6/15 (40%) patients with HER2-low/HR− breast cancer exhibited an objective response. Although not sufficient to postulate that novel anti-HER2 ADCs could be more appropriate in HR− versus HR+ HER2-low tumors, these data suggest the important role of HR status also within the HER2-low group. Taken together, our findings overall strengthen the ones previously published [12] and might be particularly useful for future therapeutic developments in patients with breast cancer.

In addition, in order to evaluate the impact of HER2-low status on prognosis, we presented survival analyses comparing HER2-low to the other BC subtypes. We did not observe any significant difference in terms of DFI, PFI, and OS when comparing HER2-low tumors with non-HER-low BCs, and this is reassuring for clinical practice, considering that patients with HER2-low BC likely did not receive any HER2-targeting therapy. However, these prognostic data are in apparent contrast with other retrospective data in the literature [31,32] and should be read with caution and considered only as exploratory, due to the retrospective design of our analysis and to the lack of adequate information about treatment received. Interestingly, HER2-low/HR+ tumors presented better DFI and PFI compared to HER2-low/HR− BC, and this further underlines the heterogeneity existing within the HER2-low group.

For the purpose of survival analyses, we used data from the integrated the TCGA Pan-Cancer Clinical Data Resource, a standardized dataset developed by Liu et al. [19], aiming to ensure proper use of clinical data in association with genomic features reported in TCGA dataset. Indeed, the relatively short follow-up and the incomplete annotation of patient outcomes as well as of treatment data for each sample have been reported as limitations of the TCGA dataset [33,34]. According to the recommendations provided by Liu et al. about the use of clinical outcome’s endpoints in each type of disease reported in TCGA dataset, both PFI and DFI are recommended for use when analyzing breast cancer data. On the contrary, OS should be used with caution, due to the relatively short follow-up (median follow-up: 27.7 months). Nonetheless, OS represents an important endpoint, being free from any ambiguity in the definition of an OS event. Therefore, in the present study, we included OS analysis, which should be interpreted with caution, due to the above-mentioned limitation.

Our study has some other limitations: (i) the retrospective design, with associated potential biases; (ii) a high number of cases presented incomplete information regarding HER2, ER, and PgR status. This lack of data affected the definition of the luminal and TNBC groups we used in the present study, although we performed subsequent analysis to ensure the reliability of data included in our analysis, and further highlights the importance of annotating clinical data with standardized procedures in genomics studies; (iii) different criteria for ER, PgR, and HER2 evaluation could be used, with no central confirmation of pathological assessment performed. For instance, most of the tumors ER and PgR status considered to be negative had IHC expression scores <10%, although the most recent guidelines recommend considering tumor samples with 1% or more nuclei positive as ER-positive [35]. In TCGA HER2 status was not defined according to the current ASCO/CAP guidelines [6], and since the definition of the HER2 status had evolved in recent years, it will further have a role in the precise identification and characterization of HER2-low tumors; (iv) for the purpose of this paper, we limited the analyses to the evaluation of PAM50 subtypes, as well as *ERBB2* and *ESR1* expression levels, while a broader analysis of gene expression profiles could provide additional information on HER2-low tumors; (v) the incomplete data available in the TCGA dataset about treatments received, which means the prognostic data should be interpreted with caution.

On the contrary, our study has some strengths. First, we used a homogeneous cohort of primary BCs extracted from a single dataset: focusing on primary BC samples allowed us to limit the variability existing between primary tumor and metastases [36,37,38]. Second, we provided a comprehensive analysis of the impact of HER2-low status on three different clinical outcomes, based on the recommendations provided in the TCGA CDR, a standardized dataset with a transparent derivation of clinical outcome endpoints and with resolution of quality concerns.

Our exploratory data can be hypothesis-generating for further studies, as it will be interesting to verify how PAM50 subtypes or *ERBB2* expression levels could impact treatment response (for instance in relation to the role of the HER2-targeted part of the ADCs in HER2-low tumors with higher *ERBB2* expression, with a HER2-enriched PAM50 subtype or according to HR status) in these breast cancer subtypes [39].

## 5. Conclusions

This characterization of the PAM50 intrinsic subtypes within HER2-low primary BC underlines the heterogeneity of this subtype reflected on clinical outcome. HER2-low/HR+ and HER2-low/HR− BCs showed a higher proportion of HER2-enriched intrinsic subtype as well as higher *ERBB2* expression levels compared to HER2−/HR+ and HER2−/HR− subtypes, respectively. This finding is consistent with previous data, and further supports the investigation of new treatment strategies in the HER2-low category. Finally, the heterogeneity in terms of PAM50 subtypes and prognosis highlights the importance of considering HR status in the HER2-low category.

## Figures and Tables

**Table 1:** Characteristics of samples included in the analysis, according to availability of IHC/FISH, PAM50, RNAseq and survival data.

Breast Cancer Subtypes	Available IHC/FISH Data *N* (%) [16]	Available PAM50 Data *N* (%) [16]	Available RNAseq Data *N* (%) [16]	Available Survival Data *N* (%) [19]
All	804 (100)	789 (100)	799 (100)	804 (100)
HER2-low/HR+	336 (41.8)	331 (42.0)	335 (41.9)	336 (41.8)
HER2-low/HR−	74 (9.2)	73 (9.2)	73 (9.1)	74 (9.2)
HER2+/HR−	32 (4.0)	32 (4.0)	32 (4.0)	32 (4.0)
HER2+/HR+	101 (12.5)	96 (12.2)	101 (12.6)	101 (12.5)
HER2−/HR+	197 (24.5)	193 (24.5)	194 (24.3)	197 (24.5)
HER2−/HR−	64 (8.0)	64 (8.1)	64 (8.0)	64 (8.0)

Abbreviations: HR+/−: hormone receptor positive/negative.

**Table 2:** Distribution of PAM50 subtypes according to clinical subgroups.

PAM50 Subtypes	Clinical Breast Cancer Subtypes						*p*-Value
	HER2−/HR+ *N* (%)	HER2-Low/HR+ *N* (%)	HER2+/HR+ *N* (%)	HER2−/HR− *N* (%)	HER2-Low/HR− *N* (%)	HER2+/HR− *N* (%)	
Basal-like	9 (4.7)	12 (3.6)	2 (2.1)	58 (90.6)	56 (76.7)	2 (6.2)	<0.001
HER2-E	1 (0.5)	4 (1.2)	20 (20.8)	1 (1.6)	10 (13.7)	29 (90.6)	
Luminal A	135 (69.9)	218 (65.9)	45 (46.9)	2 (3.1)	1 (1.4)	0 (0)	
Luminal B	41 (21.2)	84 (25.4)	28 (29.2)	1 (1.6)	0 (0)	0 (0)	
Normal-like	7 (3.6)	13 (3.9)	1 (1.0)	2 (3.1)	6 (8.2)	1 (3.1)	

Abbreviations: HER2-E: HER2-enriched, HR+/−: hormone receptor positive/negative.

## Data Availability

The breast cancer TCGA dataset analyzed in this study is publicly available in the GDC Data Portal (https://portal.gdc.cancer.gov/ (accessed on 15 October 2020)).

## Supplementary Materials

The following are available online at https://www.mdpi.com/article/10.3390/cancers13112824/s1, Figure S1: STROBE flow-chart resuming the patient selection process. Figure S2: *ERBB2* gene expression levels according to HER2 IHC score in HER2-low/HR+ versus HER2−/HR+ tumors (left plot) and in HER2-low/HR− versus HER2−/HR− tumors (right plot). Figure S3: Correlation between *ERBB2* and *ESR1* gene expression levels according to HER2 status (i.e., positive, low and negative) in the whole population, in the HR+ and HR− subgroups. Table S1: Primary breast tumors included in the present study (*n* = 804). Table S2: Treatments received by all subjects and according to breast cancer subtype, as available in the TCGA dataset [13].

## References

[1] Mendes D., Alves C., Afonso N., Cardoso F., Passos-Coelho J.L., Costa L., Andrade S., Batel-Marques F. (2015). The benefit of HER2-targeted therapies on overall survival of patients with metastatic HER2-positive breast cancer-a systematic review. Breast Cancer Res..

[2] Viani G.A., Afonso S.L., Stefano E.J., De Fendi L.I., Soares F.V. (2007). Adjuvant trastuzumab in the treatment of her-2-positive early breast cancer: A meta-analysis of published randomized trials. BMC Cancer.

[3] Swain S.M., Miles D., Kim S.-B., Im Y.-H., Im S.-A., Semiglazov V., Ciruelos E., Schneeweiss A., Loi S., Monturus E. (2020). Pertuzumab, trastuzumab, and docetaxel for HER2-positive metastatic breast cancer (CLEOPATRA): End-of-study results from a double-blind, randomised, placebo-controlled, phase 3 study. Lancet Oncol..

[4] Slamon D.J., Clark G.M., Wong S.G., Levin W.J., Ullrich A., McGuire W.L. (1987). Human breast cancer: Correlation of relapse and survival with amplification of the HER-2/neu oncogene. Science.

[5] Ross J.S., Fletcher J.A. (1998). The HER-2/neu oncogene in breast cancer: Prognostic factor, predictive factor, and target for therapy. Stem Cells.

[6] Wolff A.C., Hammond M.E.H., Allison K.H., Harvey B.E., Mangu P.B., Bartlett J.M.S., Bilous M., Ellis I.O., Fitzgibbons P., Hanna W. (2018). Human Epidermal Growth Factor Receptor 2 Testing in Breast Cancer: American Society of Clinical Oncology/College of American Pathologists Clinical Practice Guideline Focused Update. Arch. Pathol. Lab. Med..

[7] Tarantino P., Hamilton E., Tolaney S.M., Cortes J., Morganti S., Ferraro E., Marra A., Viale G., Trapani D., Cardoso F. (2020). HER2-Low Breast Cancer: Pathological and Clinical Landscape. J. Clin. Oncol..

[8] Marchiò C., Annaratone L., Marques A., Casorzo L., Berrino E., Sapino A. (2020). Evolving concepts in HER2 evaluation in breast cancer: Heterogeneity, HER2-low carcinomas and beyond. Semin. Cancer Biol..

[9] Riecke K., Witzel I. (2020). Targeting the Human Epidermal Growth Factor Receptor Family in Breast Cancer beyond HER2. Breast Care (Basel).

[10] Eiger D., Agostinetto E., Saúde-Conde R., de Azambuja E. (2021). The Exciting New Field of HER2-Low Breast Cancer Treatment. Cancers (Basel).

[11] Fehrenbacher L., Cecchini R.S., Geyer C.E., Rastogi P., Costantino J.P., Atkins J.N., Crown J.P., Polikoff J., Boileau J.-F., Provencher L. (2019). NSABP B-47/NRG Oncology Phase III Randomized Trial Comparing Adjuvant Chemotherapy With or Without Trastuzumab in High-Risk Invasive Breast Cancer Negative for HER2 by FISH and With IHC 1+ or 2+. J. Clin. Oncol..

[12] Schettini F., Chic N., Brasó-Maristany F., Paré L., Pascual T., Conte B., Martínez-Sáez O., Adamo B., Vidal M., Barnadas E. (2021). Clinical, pathological, and PAM50 gene expression features of HER2-low breast cancer. NPJ Breast Cancer.

[13] Prat A., Carey L.A., Adamo B., Vidal M., Tabernero J., Cortés J., Parker J.S., Perou C.M., Baselga J. (2014). Molecular features and survival outcomes of the intrinsic subtypes within HER2-positive breast cancer. J. Natl. Cancer Inst..

[14] Prat A., Pineda E., Adamo B., Galván P., Fernández A., Gaba L., Díez M., Viladot M., Arance A., Muñoz M. (2015). Clinical implications of the intrinsic molecular subtypes of breast cancer. Breast.

[15] Prat A., Chaudhury A., Solovieff N., Paré L., Martinez D., Chic N., Martínez-Sáez O., Brasó-Maristany F., Lteif A., Taran T. (2021). Correlative biomarker analysis of intrinsic subtypes and efficacy across the MONALEESA Phase III studies. J. Clin. Oncol..

[16] Colaprico A., Silva T.C., Olsen C., Garofano L., Cava C., Garolini D., Sabedot T.S., Malta T.M., Pagnotta S.M., Castiglioni I. (2016). TCGAbiolinks: An R/Bioconductor package for integrative analysis of TCGA data. Nucleic Acids Res..

[17] Wolff A.C., Hammond M.E.H., Hicks D.G., Dowsett M., McShane L.M., Allison K.H., Allred D.C., Bartlett J.M.S., Bilous M., Fitzgibbons P. (2014). Recommendations for human epidermal growth factor receptor 2 testing in breast cancer: American Society of Clinical Oncology/College of American Pathologists clinical practice guideline update. Arch. Pathol. Lab. Med..

[18] Parker J.S., Mullins M., Cheang M.C.U., Leung S., Voduc D., Vickery T., Davies S., Fauron C., He X., Hu Z. (2009). Supervised risk predictor of breast cancer based on intrinsic subtypes. J. Clin. Oncol..

[19] Liu J., Lichtenberg T., Hoadley K.A., Poisson L.M., Lazar A.J., Cherniack A.D., Kovatich A.J., Benz C.C., Levine D.A., Lee A.V. (2018). An Integrated TCGA Pan-Cancer Clinical Data Resource to Drive High-Quality Survival Outcome Analytics. Cell.

[20] Modi S., Park H., Murthy R.K., Iwata H., Tamura K., Tsurutani J., Moreno-Aspitia A., Doi T., Sagara Y., Redfern C. (2020). Antitumor Activity and Safety of Trastuzumab Deruxtecan in Patients With HER2-Low–Expressing Advanced Breast Cancer: Results From a Phase Ib Study. J. Clin. Oncol..

[21] Banerji U., van Herpen C.M.L., Saura C., Thistlethwaite F., Lord S., Moreno V., Macpherson I.R., Boni V., Rolfo C., de Vries E.G.E. (2019). Trastuzumab duocarmazine in locally advanced and metastatic solid tumours and HER2-expressing breast cancer: A phase 1 dose-escalation and dose-expansion study. Lancet Oncol..

[22] Clifton G.T., Hale D., Vreeland T.J., Hickerson A.T., Litton J.K., Alatrash G., Murthy R.K., Qiao N., Philips A.V., Lukas J.J. (2020). Results of a Randomized Phase IIb Trial of Nelipepimut-S + Trastuzumab versus Trastuzumab to Prevent Recurrences in Patients with High-Risk HER2 Low-Expressing Breast Cancer. Clin. Cancer Res..

[23] Mittendorf E.A., Lu B., Melisko M., Price Hiller J., Bondarenko I., Brunt A.M., Sergii G., Petrakova K., Peoples G.E. (2019). Efficacy and Safety Analysis of Nelipepimut-S Vaccine to Prevent Breast Cancer Recurrence: A Randomized, Multicenter, Phase III Clinical Trial. Clin. Cancer Res..

[24] Iwata H., Tamura K., Doi T., Tsurutani J., Modi S., Park H., Krop I.E., Sagara Y., Redfern C.H., Murthy R.K. (2018). Trastuzumab deruxtecan (DS-8201a) in subjects with HER2-expressing solid tumors: Long-term results of a large phase 1 study with multiple expansion cohorts. J. Clin. Oncol..

[25] Rinnerthaler G., Gampenrieder S.P., Greil R. (2019). HER2 directed antibody-drug-conjugates beyond T-DM1 in breast cancer. Int. J. Mol. Sci..

[26] Koboldt D.C., Fulton R.S., McLellan M.D., Schmidt H., Kalicki-Veizer J., McMichael J.F., Fulton L.L., Dooling D.J., Ding L., Mardis E.R. (2012). Comprehensive molecular portraits of human breast tumours. Nature.

[27] Razavi P., Chang M.T., Xu G., Bandlamudi C., Ross D.S., Vasan N., Cai Y., Bielski C.M., Donoghue M.T.A., Jonsson P. (2018). The Genomic Landscape of Endocrine-Resistant Advanced Breast Cancers. Cancer Cell.

[28] Prat A., Pascual T., De Angelis C., Gutierrez C., Llombart-Cussac A., Wang T., Cortés J., Rexer B., Paré L., Forero A. (2020). HER2-Enriched Subtype and ERBB2 Expression in HER2-Positive Breast Cancer Treated with Dual HER2 Blockade. J. Natl. Cancer Inst..

[29] Fumagalli D., Venet D., Ignatiadis M., Azim H.A.J., Maetens M., Rothé F., Salgado R., Bradbury I., Pusztai L., Harbeck N. (2017). RNA Sequencing to Predict Response to Neoadjuvant Anti-HER2 Therapy: A Secondary Analysis of the NeoALTTO Randomized Clinical Trial. JAMA Oncol..

[30] Pinhel I., Hills M., Drury S., Salter J., Sumo G., A’Hern R., Bliss J.M., Sestak I., Cuzick J., Barrett-Lee P. (2012). ER and HER2 expression are positively correlated in HER2 non-overexpressing breast cancer. Breast Cancer Res..

[31] Rossi V., Sarotto I., Maggiorotto F., Berchialla P., Kubatzki F., Tomasi N., Redana S., Martinello R., Valabrega G., Aglietta M. (2012). Moderate immunohistochemical expression of HER-2 (2+) without HER-2 gene amplification is a negative prognostic factor in early breast cancer. Oncologist.

[32] Eggemann H., Ignatov T., Burger E., Kantelhardt E.J., Fettke F., Thomssen C., Costa S.D., Ignatov A. (2015). Moderate HER2 expression as a prognostic factor in hormone receptor positive breast cancer. Endocr. Relat. Cancer.

[33] Hoadley K.A., Yau C., Wolf D.M., Cherniack A.D., Tamborero D., Ng S., Leiserson M.D.M., Niu B., McLellan M.D., Uzunangelov V. (2014). Multiplatform analysis of 12 cancer types reveals molecular classification within and across tissues of origin. Cell.

[34] Huo D., Hu H., Rhie S.K., Gamazon E.R., Cherniack A.D., Liu J., Yoshimatsu T.F., Pitt J.J., Hoadley K.A., Troester M. (2017). Comparison of Breast Cancer Molecular Features and Survival by African and European Ancestry in The Cancer Genome Atlas. JAMA Oncol..

[35] Allison K.H., Hammond M.E.H., Dowsett M., McKernin S.E., Carey L.A., Fitzgibbons P.L., Hayes D.F., Lakhani S.R., Chavez-MacGregor M., Perlmutter J. (2020). Estrogen and Progesterone Receptor Testing in Breast Cancer: ASCO/CAP Guideline Update. J. Clin. Oncol..

[36] Kalinowski L., Saunus J.M., McCart Reed A.E., Lakhani S.R. (2019). Breast Cancer Heterogeneity in Primary and Metastatic Disease. Adv. Exp. Med. Biol..

[37] Cejalvo J.M., de Dueñas E.M., Galván P., García-Recio S., Gasión O.B., Paré L., Antolín S., Martinello R., Blancas I., Adamo B. (2017). Intrinsic Subtypes and Gene Expression Profiles in Primary and Metastatic Breast Cancer. Cancer Res..

[38] Aftimos P.G., e Oliveira A.A.D.M., Hilbers F., Venet D., Vingiani A., Nili Gal Yam E., Martinez J.L., Ndozeng J., Irrthum A., Piccart M. (2019). First report of AURORA, the breast international group (BIG) molecular screening initiative for metastatic breast cancer (MBC) patients (pts). Ann. Oncol..

[39] Griguolo G., Brasó-Maristany F., González-Farré B., Pascual T., Chic N., Saurí T., Kates R., Gluz O., Martínez D., Paré L. (2020). ERBB2 mRNA Expression and Response to Ado-Trastuzumab Emtansine (T-DM1) in HER2-Positive Breast Cancer. Cancers (Basel).

