# Supplementary material for: HER2-Low Breast Cancer: Molecular Characteristics and Prognosis
Source: Cancers (Basel). 2021 Jun 5;13(11):2824. doi: 10.3390/cancers13112824 (PMC8201345; doi:10.3390/cancers13112824)
Supplement: Supplementary file 1 [file cancers-13-02824-s001.zip › cancers-1214378-supplementary.pdf]

cancers MDPI

Article

Supplementary Materials: HER2-Low Breast Cancer: Molecular Characteristics and Prognosis

Elisa Agostinetto, Mattia Rediti, Danai Fimereli, Véronique Debien, Martine Piccart, Philippe Aftimos, Christos Sotiriou and Evandro de Azambuja

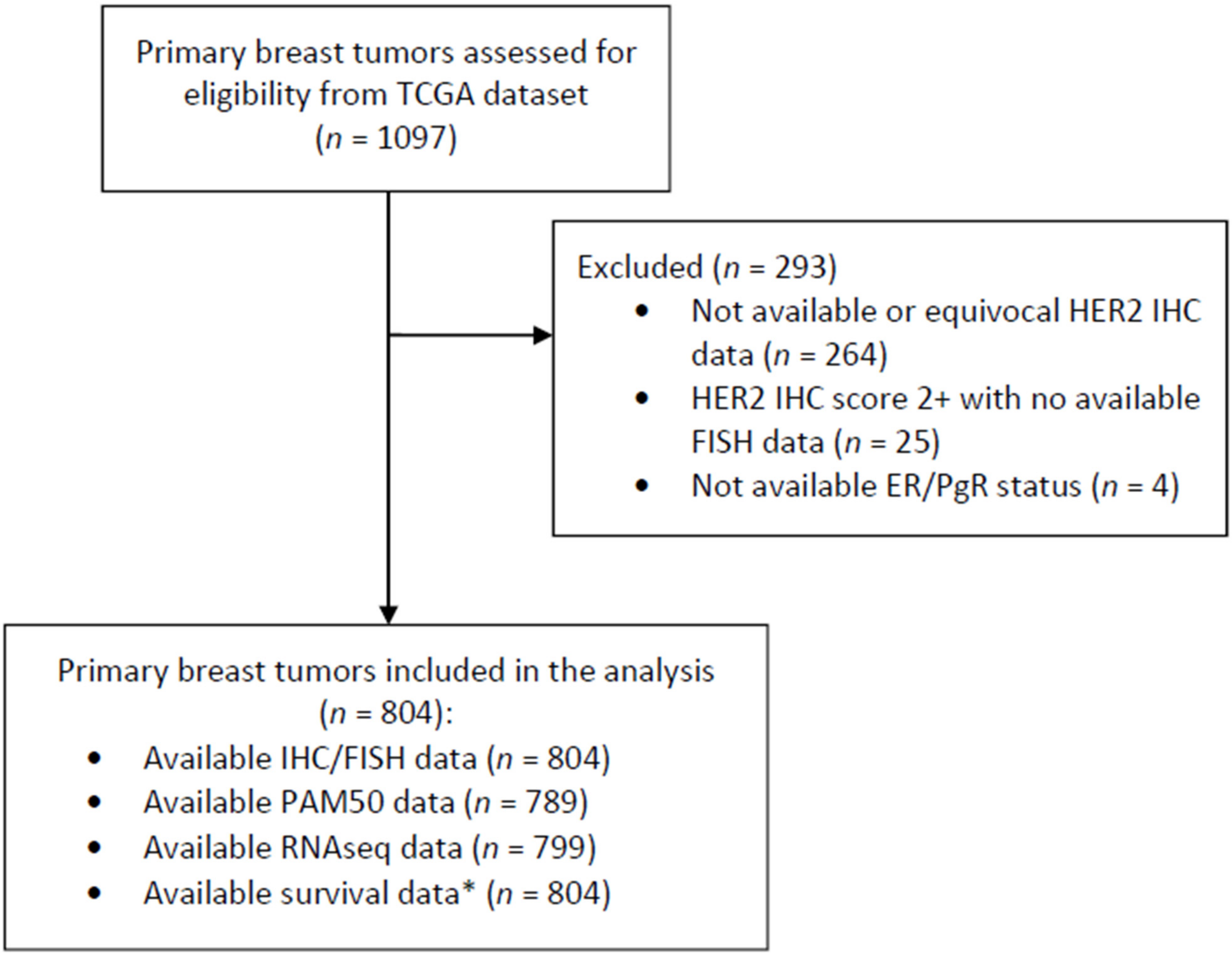

Figure S1. STROBE flow-chart resuming the patient selection process. * Survival data were obtained from the TCGA Pan-Cancer Clinical Data Resource, developed by Liu et al. [15]. Abbreviations: TCGA: The Cancer Genome Atlas, IHC: immunohistochemistry, FISH: fluorescent in situ hybridization, ER: estrogen receptors, PgR: progesterone receptor.

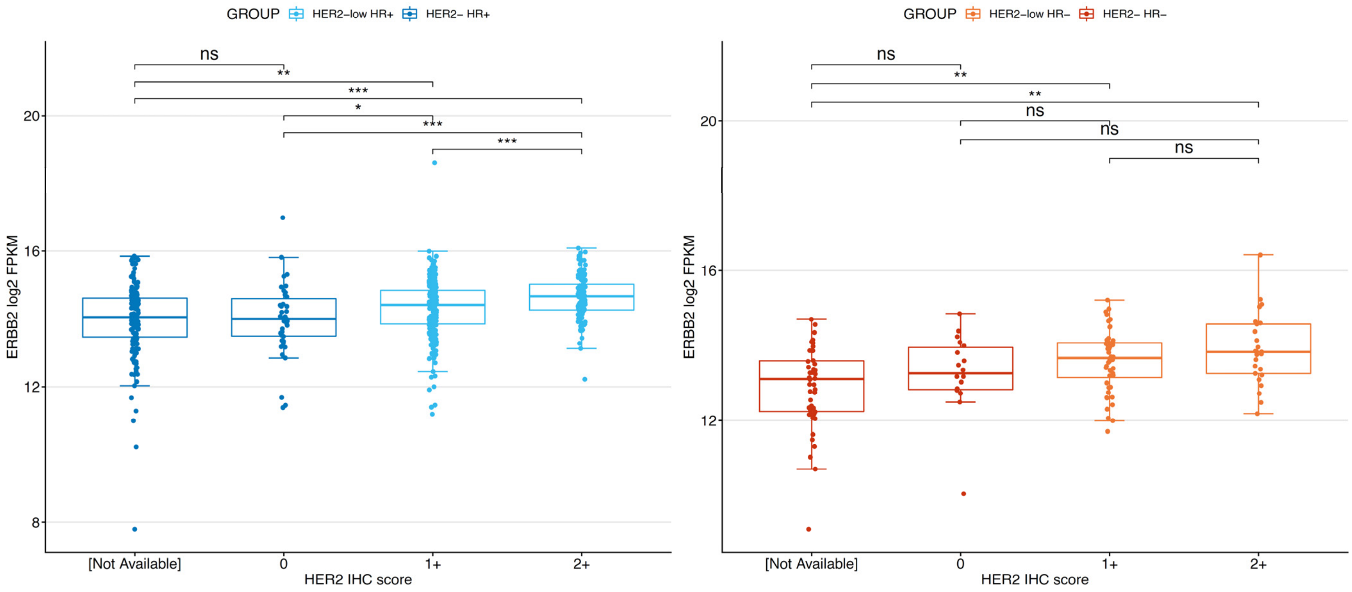

Figure S2. *ERBB2* gene expression levels according to HER2 IHC score in HER2-low/HR+ versus HER2−/HR+ tumors (left plot) and in HER2-low/HR− versus HER2−/HR− tumors (right plot). Statistical differences were assessed using a two-sided Mann–Whitney U-test followed by Benjamin and Hochberg correction for multiple testing. Abbreviations: IHC: immunohistochemistry, ns = not significant; * = adjusted *p*-value < 0.05 and ≥ 0.01; ** = adjusted *p*-value < 0.01 and ≥ 0.001; *** = adjusted *p*-value < 0.001.

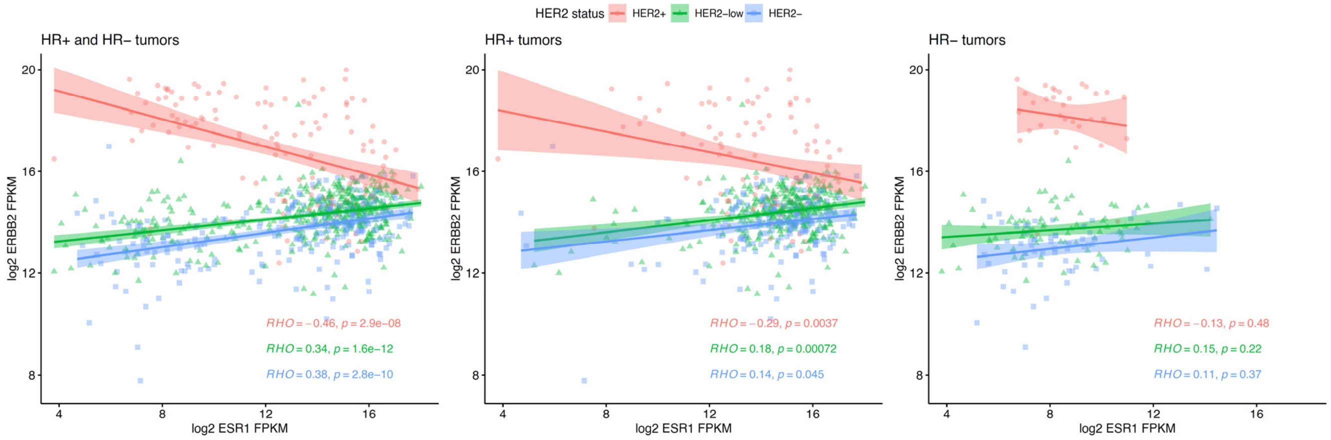

Figure S3. Correlation between *ERBB2* and *ESR1* gene expression levels according to HER2 status (i.e., positive, low and negative) in the whole population, in the HR+ and HR− subgroups. RHO is the Spearman correlation (color-coded according to HER2 status). Red, green, and blue colored regions indicate the 95% confidence interval for each regression line. Abbreviations: HR: hormone receptor.

Table S1. Primary breast tumors included in the present study (n = 804).

GROUP	her2_ihc_score	her2_fish_status	her2_status_by_ihc	er_status_by_ihc	pr_status_by_ihc	*N*
HER2−/HR−	[Not Available]	[Not Evaluated]	Negative	Negative	Negative	42
HER2−/HR−	0	[Not Evaluated]	Negative	Negative	Negative	13
HER2−/HR−	[Not Available]	Negative	Negative	Negative	Negative	4
HER2−/HR−	0	Negative	Negative	Negative	Negative	5
HER2−/HR+	[Not Available]	[Not Evaluated]	Negative	Positive	Indeterminate	1
HER2−/HR+	[Not Available]	[Not Evaluated]	Negative	Positive	Negative	24
HER2−/HR+	0	[Not Evaluated]	Negative	Positive	Negative	5
HER2−/HR+	[Not Available]	Negative	Negative	Positive	Negative	5
HER2−/HR+	0	Negative	Negative	Positive	Negative	1
HER2−/HR+	[Not Available]	[Not Evaluated]	Negative	Negative	Positive	4

HER2−/HR+	[Not Available]	Negative	Negative	Negative	Positive	1
HER2−/HR+	[Not Available]	[Not Evaluated]	Negative	Positive	Positive	98
HER2−/HR+	0	[Not Evaluated]	Negative	Positive	Positive	25
HER2−/HR+	[Not Available]	Negative	Negative	Positive	Positive	21
HER2−/HR+	0	Negative	Negative	Positive	Positive	12
HER2+/HR+	3+	Positive	Positive	Indeterminate (considered positive as column "er_status_ihc_Percent_positive " = 20 29%	Negative	1
HER2+/HR+	3+	Negative	Equivocal	Positive	Negative	1
HER2+/HR+	2+	Positive	Equivocal	Positive	Negative	5
HER2+/HR+	3+	[Not Evaluated]	Positive	Positive	Negative	6
HER2+/HR+	3+	Negative	Positive	Positive	Negative	1
HER2+/HR+	[Not Available]	Positive	Positive	Positive	Negative	2
HER2+/HR+	2+	Positive	Positive	Positive	Negative	3
HER2+/HR+	3+	Positive	Positive	Positive	Negative	5
HER2+/HR+	2+	Positive	Equivocal	Negative	Positive	1
HER2+/HR+	3+	[Not Evaluated]	Positive	Negative	Positive	1
HER2+/HR+	2+	Positive	Equivocal	Positive	Positive	11
HER2+/HR+	3+	Negative	Indeterminate	Positive	Positive	1
HER2+/HR+	2+	Positive	Indeterminate	Positive	Positive	2
HER2+/HR+	3+	[Not Evaluated]	Negative	Positive	Positive	1
HER2+/HR+	3+	[Not Available]	Positive	Positive	Positive	3
HER2+/HR+	3+	[Not Evaluated]	Positive	Positive	Positive	28
HER2+/HR+	3+	Negative	Positive	Positive	Positive	1
HER2+/HR+	[Not Available]	Positive	Positive	Positive	Positive	4
HER2+/HR+	2+	Positive	Positive	Positive	Positive	12
HER2+/HR+	3+	Positive	Positive	Positive	Positive	12
HER2+/HR−	3+	[Not Evaluated]	[Not Evaluated]	Negative	Negative	1
HER2+/HR−	2+	Positive	Equivocal	Negative	Negative	2
HER2+/HR−	3+	[Not Evaluated]	Negative	Negative	Negative	1
HER2+/HR−	3+	[Not Evaluated]	Positive	Negative	Negative	21
HER2+/HR−	3+	Negative	Positive	Negative	Negative	1
HER2+/HR−	[Not Available]	Positive	Positive	Negative	Negative	1
HER2+/HR−	3+	Positive	Positive	Negative	Negative	5
HER2-low/HR−	1+	Negative	Equivocal	Negative	Negative	1
HER2-low/HR−	2+	Negative	Equivocal	Negative	Negative	24
HER2-low/HR−	1+	[Not Available]	Negative	Negative	Negative	2
HER2-low/HR−	1+	[Not Evaluated]	Negative	Negative	Negative	33
HER2-low/HR−	1+	Negative	Negative	Negative	Negative	9
HER2-low/HR−	1+	Positive	Negative	Negative	Negative	2
HER2-low/HR−	1+	[Not Evaluated]	Positive	Negative	Negative	2
HER2-low/HR−	2+	Negative	Positive	Negative	Negative	1
HER2-low/HR+	1+	[Not Evaluated]	Negative	Positive	Indeterminate	1
HER2-low/HR+	2+	Negative	Equivocal	Positive	Negative	11
HER2-low/HR+	1+	[Not Evaluated]	Negative	Positive	Negative	17
HER2-low/HR+	1+	Negative	Negative	Positive	Negative	8
HER2-low/HR+	1+	Positive	Negative	Positive	Negative	1
HER2-low/HR+	1+	[Not Evaluated]	Positive	Positive	Negative	2
HER2-low/HR+	1+	Negative	Equivocal	Negative	Positive	1
HER2-low/HR+	2+	Negative	Equivocal	Negative	Positive	2
HER2-low/HR+	1+	[Not Evaluated]	Negative	Negative	Positive	3
HER2-low/HR+	1+	Negative	Equivocal	Positive	Positive	2
HER2-low/HR+	2+	Negative	Equivocal	Positive	Positive	92
HER2-low/HR+	1+	[Not Evaluated]	Indeterminate	Positive	Positive	1

HER2-low/HR+	2+	Negative	Indeterminate	Positive	Positive	2
HER2-low/HR+	1+	[Not Evaluated]	Negative	Positive	Positive	125
HER2-low/HR+	1+	Negative	Negative	Positive	Positive	55
HER2-low/HR+	2+	Negative	Negative	Positive	Positive	1
HER2-low/HR+	1+	[Not Evaluated]	Positive	Positive	Positive	7
HER2-low/HR+	2+	Negative	Positive	Positive	Positive	5

Table S2. Treatments received by all subjects and according to breast cancer subtype, as available in the TCGA dataset [13].

	**All Subjects** ***N* (%)**	**Available Treatment Data** ***N* (%)**	**Anti-HER2 Treatment** ***N***
**All**	804 (100)	628 (78.1)	59
**HER2-low/HR+**	336	279	0
**HER2-low/HR−**	74	61	1
**HER2+/HR−**	32	23	16
**HER2+/HR+**	101	75	40
**HER2−/HR+**	197	144	2
**HER2−/HR−**	64	46	0
